# Supplementary figures and images for: A minisatellite-based MLVA for deciphering the global epidemiology of the bacterial cassava pathogen Xanthomonas phaseoli pv. manihotis
Source: PLoS One. 2023 May 11;18(5):e0285491. doi: 10.1371/journal.pone.0285491 (PMC10174486; doi:10.1371/journal.pone.0285491)

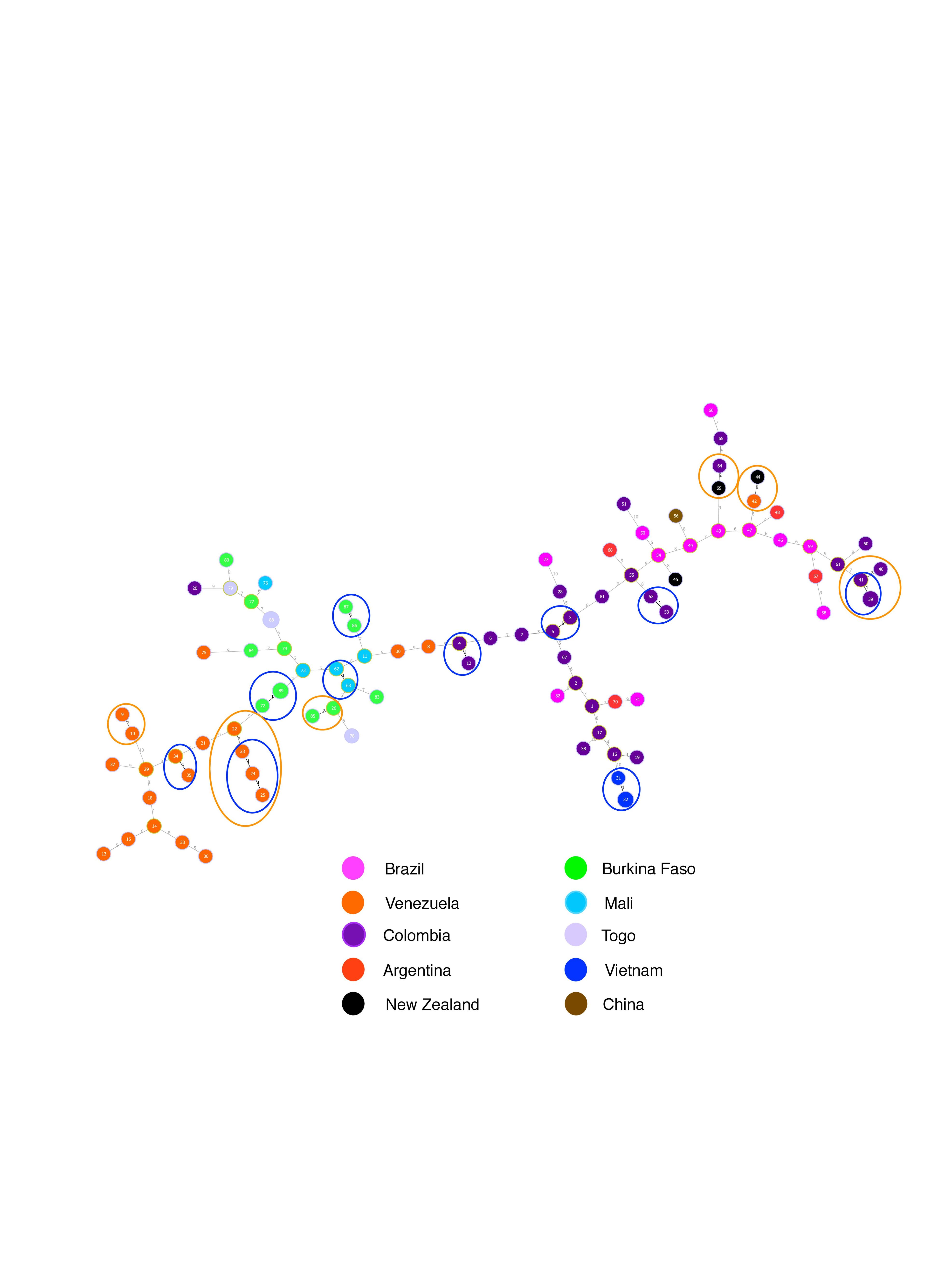

Supplement: S1 Fig — Colors indicate the origin of the haplotype, and the circle size indicates the number of strains of each haplotype. Blue circles indicate clonal complexes and orange circles indicate groups of double locus variants. Numbers indicate the number of loci variants between haplotypes. (TIF) [file pone.0285491.s001.tif]

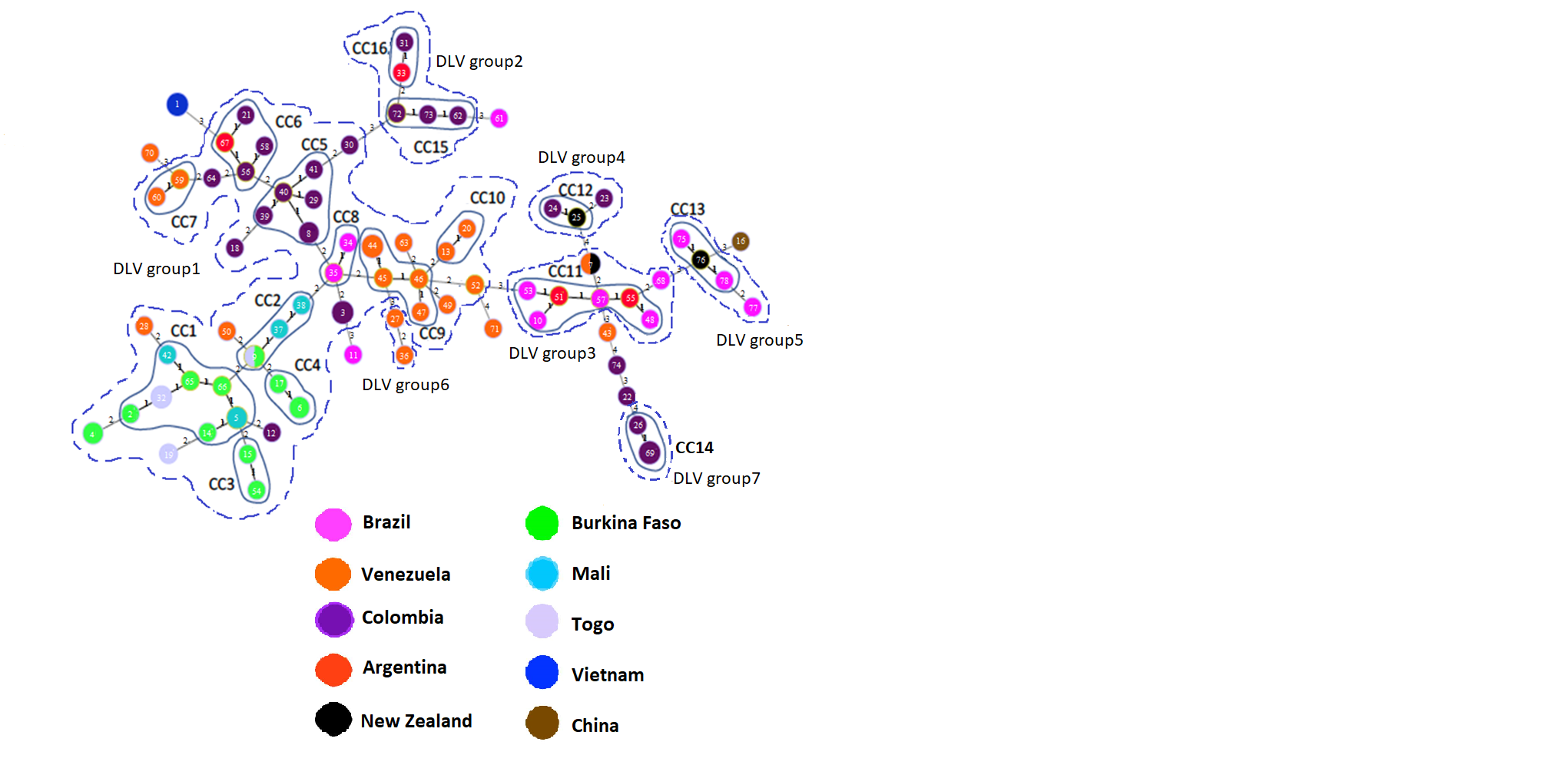

Supplement: S2 Fig — Colors indicate the origin of the haplotype, and the circle size is relative to the number of strains of each haplotype. The solid lines represent clonal complexes (CCs) grouping single locus variants and dotted lines group up to double locus variants. Numbers indicate the number of loci variants between haplotypes. (TIF) [file pone.0285491.s002.tif]

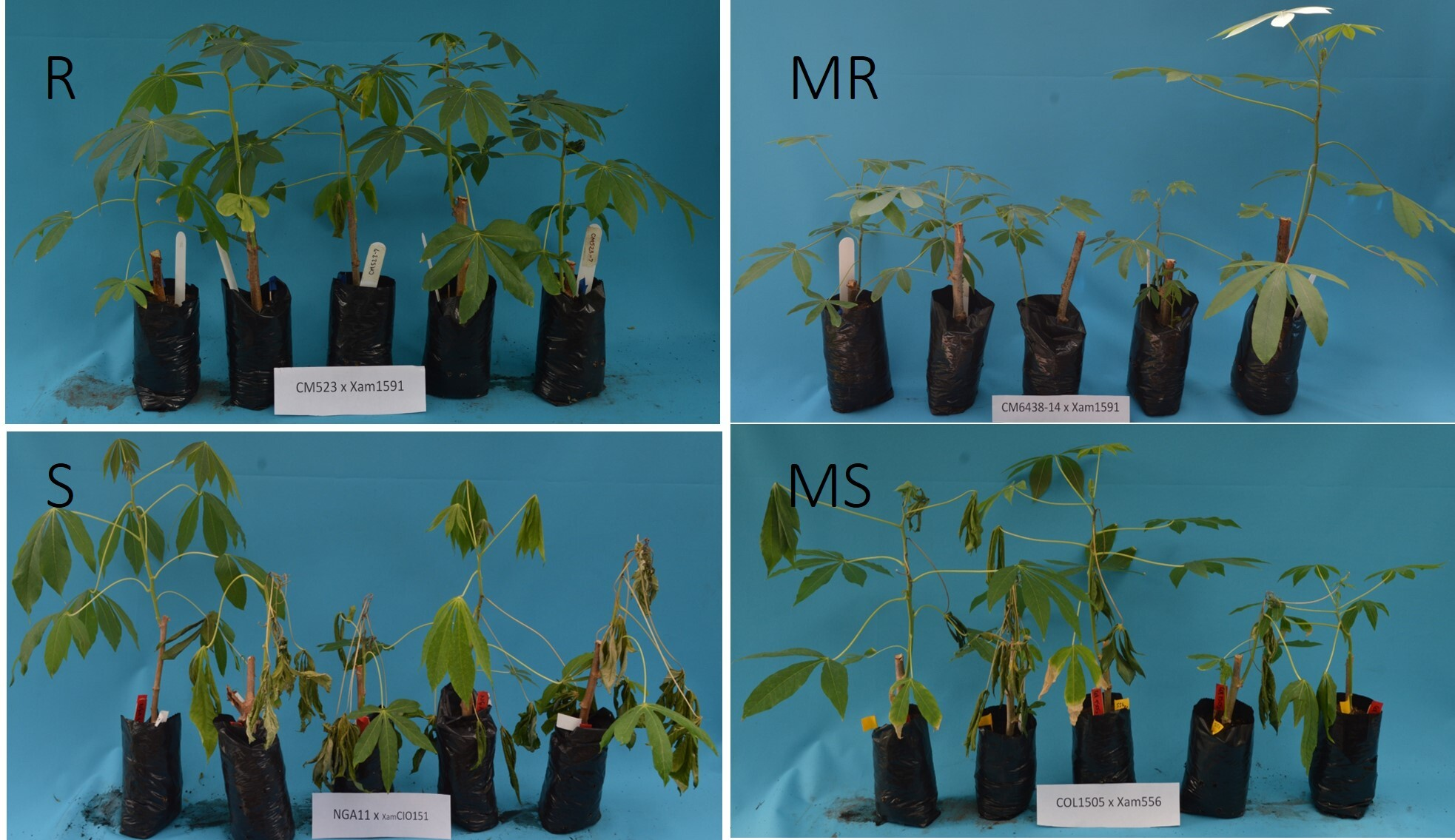

Supplement: S3 Fig — (TIF) [file pone.0285491.s003.tif]

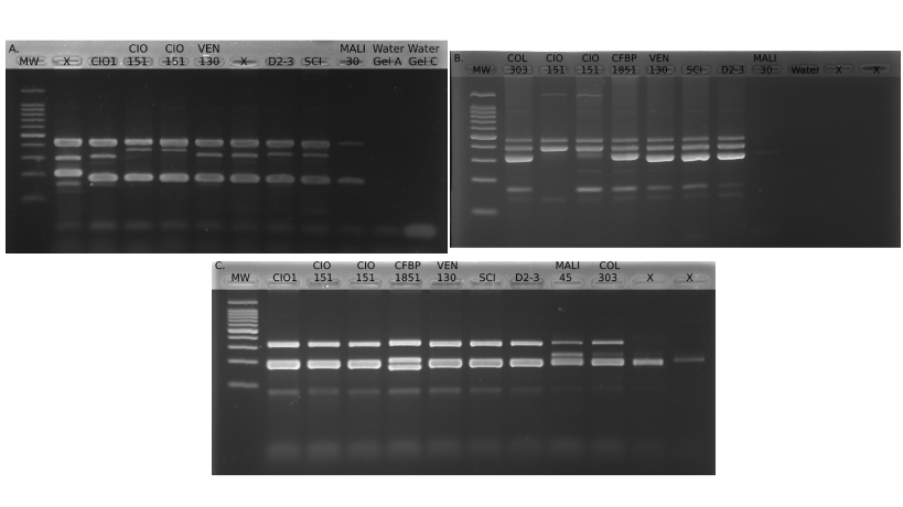

Supplement: S4 Fig — (TIF) [file pone.0285491.s004.tif]
