# Supplementary material for: A minisatellite-based MLVA for deciphering the global epidemiology of the bacterial cassava pathogen Xanthomonas phaseoli pv. manihotis
Source: PLoS One. 2023 May 11;18(5):e0285491. doi: 10.1371/journal.pone.0285491 (PMC10174486; doi:10.1371/journal.pone.0285491)
Supplement: S1 Table — (DOCX) [file pone.0285491.s005.docx]

|  |  |  |  | Haplotype | | |  |
| --- | --- | --- | --- | --- | --- | --- | --- |
| strain | country | year | region | MLVA-8 | MLVA-12 | MLVA-14 | MLVA-12 DAPC cluster |
| CIAT1241 | Argentina | 1984 | nd ^a^ | 15 | 58 | 48 | 2 |
| INTA1 | Argentina | ND | nd | 13 | 31 | 68 | 7 |
| INTA10 | Argentina | ND | nd | 15 | 55 | 57 | 2 |
| INTA5 | Argentina | ND | nd | 3 | 67 | 70 | 1 |
| CIAT1117 | Brazil | 1974 | nd | 7 | 61 | 66 | 7 |
| CIAT1120 | Brazil | 1974 | nd | 1 | 34 | 71 | 1 |
| CIO337 | Brazil | 1996 | Sao Paulo | 27 | 53 | 59 | 2 |
| CIO338 | Brazil | 1996 | Sao Paulo | 17 | 78 | 54 | 6 |
| CIO339 | Brazil | 1996 | Sao Paulo | 15 | 57 | 46 | 2 |
| CIO340 | Brazil | 1996 | Sao Paulo | 1 | 35 | 82 | 1 |
| CIO347 | Brazil | 1996 | Mato Grosso do Sul | 15 | 56 | 47 | 2 |
| CIO349 | Brazil | 1996 | Mato Grosso do Sul | 9 | 42 | 43 | 2 |
| CIO356 | Brazil | 1995 | Sao Paulo | 30 | 75 | 49 | 6 |
| CIO358 | Brazil | 1996 | Sao Paulo | 14 | 51 | 27 | 1 |
| CIO366 | Brazil | 1995 | Sao Paulo | 17 | 77 | 50 | 6 |
| LMG777 | Brazil | 1978 | Goiani | 15 | 54 | 58 | 2 |
| P11-11 | Burkina-Faso | 2016 | Hauts-Bassins | 18 | 17 | 89 | 3 |
| P13-16 | Burkina-Faso | 2016 | Hauts-Bassins | 5 | 6 | 89 | 3 |
| P13-17 | Burkina-Faso | 2016 | Hauts-Bassins | 5 | 6 | 72 | 3 |
| P16-1 | Burkina-Faso | 2016 | Cascades | 4 | 14 | 85 | 3 |
| P16-11 | Burkina-Faso | 2016 | Cascades | 4 | 15 | 26 | 3 |
| P18-2 | Burkina-Faso | 2016 | Hauts-Bassins | 5 | 4 | 86 | 3 |
| P19-8 | Burkina-Faso | 2016 | Hauts-Bassins | 3 | 65 | 84 | 3 |
| P20-12 | Burkina-Faso | 2016 | Hauts-Bassins | 5 | 4 | 87 | 3 |
| P21-11 | Burkina-Faso | 2016 | Hauts-Bassins | 4 | 12 | 74 | 3 |
| P23-11 | Burkina-Faso | 2016 | Cascades | 2 | 9 | 77 | 3 |
| P23-13 | Burkina-Faso | 2016 | Cascades | 4 | 13 | 80 | 3 |
| P24-10 | Burkina-Faso | 2016 | Cascades | 3 | 63 | 83 | 3 |
| CIAT1135 | China | 1975 | nd | 19 | 16 | 56 | 6 |
| CFBP1851 | Colombia | 1974 | nd | 22 | 24 | 64 | 6 |
| CIAT1080 | Colombia | 1972 | nd | 16 | 72 | 55 | 7 |
| CIAT1180 | Colombia | 1977 | nd | 29 | 74 | 60 | 4 |
| CIAT1202 | Colombia | 1981 | nd | 13 | 30 | 51 | 7 |
| CIO116 | Colombia | 1995 | Eastern Plains | 8 | 2 | 39 | 4 |
| CIO121 | Colombia | 1995 | Eastern Plains | 1 | 3 | 17 | 1 |
| CIO124 | Colombia | 1995 | Eastern Plains | 24 | 32 | 40 | 4 |
| CIO129 | Colombia | 1995 | Eastern Plains | 1 | 3 | 19 | 1 |
| CIO151 | Colombia | 1995 | nd | 18 | 18 | 38 | 1 |
| CIO164 | Colombia | 1995 | Eastern Plains | 8 | 2 | 39 | 4 |
| CIO167 | Colombia | 1995 | Eastern Plains | 20 | 22 | 61 | 4 |
| CIO169 | Colombia | 1995 | Eastern Plains | 1 | 3 | 16 | 1 |
| CIO37 | Colombia | 1995 | Eastern Plains | 8 | 2 | 41 | 4 |
| ORST4 | Colombia | 1974 | nd | 21 | 23 | 65 | 6 |
| UA1092 | Colombia | 2010 | Cienaga de oro | 1 | 41 | 6 | 1 |
| UA1549 | Colombia | 2013 | Cienaga de oro | 1 | 39 | 81 | 1 |
| UA1564 | Colombia | 2014 | San Jacinto | 3 | 66 | 5 | 1 |
| UA1567 | Colombia | 2013 | Chinu | 1 | 40 | 2 | 1 |
| UA1579 | Colombia | 2013 | San Jacinto | 12 | 29 | 7 | 1 |
| UA1591 | Colombia | 2013 | Cienaga de oro | 7 | 59 | 3 | 1 |
| UA1723 | Colombia | 2014 | Cienaga de oro | 1 | 38 | 67 | 1 |
| UA1751 | Colombia | 2014 | Cienaga de oro | 3 | 68 | 28 | 1 |
| UA2162 | Colombia | 2015 | Chinu | 1 | 7 | 4 | 1 |
| UA2164 | Colombia | 2015 | Chinu | 1 | 7 | 12 | 1 |
| UA294 | Colombia | 2008 | Chinu | 16 | 73 | 52 | 7 |
| UA305 | Colombia | 2008 | Chinu | 7 | 62 | 53 | 7 |
| UA556 | Colombia | 2009 | Palmitos | 4 | 11 | 20 | 3 |
| UA681 | Colombia | 2009 | Palmitos | 10 | 21 | 1 | 1 |
| 10M | Mali | 2016 | Bamako | 1 | 36 | 76 | 3 |
| 11M | Mali | 2016 | Bamako | 2 | 48 | 11 | 3 |
| 16M | Mali | 2016 | Bamako | 4 | 5 | 62 | 3 |
| 19M | Mali | 2016 | Bamako | 4 | 5 | 63 | 3 |
| 40M | Mali | 2016 | Segou | 1 | 37 | 73 | 3 |
| CIAT1205 | New-Zealand | 1966 | nd | 23 | 25 | 69 | 6 |
| CIAT1206 | New-Zealand | 1966 | nd | 9 | 8 | 44 | 2 |
| CIAT1211 | New-Zealand | 1980 | nd | 31 | 76 | 45 | 6 |
| T1117 | Togo | ND | nd | 3 | 10 | 88 | 3 |
| T139 | Togo | ND | nd | 3 | 10 | 88 | 3 |
| T87 | Togo | ND | nd | 2 | 9 | 79 | 3 |
| X27 | Togo | 1989 | nd | 11 | 26 | 78 | 3 |
| 1942 | Venezuela | 1995 | Monagas | 2 | 50 | 75 | 3 |
| Cix1054 | Venezuela | 1995 | Monagas | 3 | 69 | 9 | 3 |
| Cix1057 | Venezuela | 1995 | Monagas | 10 | 19 | 34 | 5 |
| Cix1058 | Venezuela | 1995 | Monagas | 10 | 20 | 35 | 5 |
| Cix1066 | Venezuela | 1995 | Monagas | 14 | 52 | 36 | 5 |
| Cix1076 | Venezuela | 1995 | Monagas | 28 | 71 | 21 | 5 |
| Cix1146 | Venezuela | 1995 | Bolivar | 26 | 43 | 37 | 5 |
| Cix1155 | Venezuela | 1995 | Anzoategui | 7 | 60 | 10 | 7 |
| ORST2 | Venezuela | 1971 | Maracaibo | 9 | 8 | 42 | 2 |
| VEN001 | Venezuela | 2016 | Pelayo | 2 | 47 | 25 | 5 |
| VEN002 | Venezuela | 2016 | Pelayo | 2 | 44 | 23 | 5 |
| VEN008 | Venezuela | 2016 | Pelayo | 3 | 64 | 14 | 5 |
| VEN039 | Venezuela | 2016 | Pelayo | 2 | 44 | 24 | 5 |
| VEN040 | Venezuela | 2016 | Pelayo | 2 | 46 | 15 | 5 |
| VEN048 | Venezuela | 2016 | Pelayo | 2 | 44 | 22 | 5 |
| VEN055 | Venezuela | 2016 | Pelayo | 11 | 27 | 29 | 5 |
| VEN060 | Venezuela | 2016 | Pelayo | 2 | 49 | 13 | 5 |
| VEN078 | Venezuela | 2016 | Fuerte-Paramacay | 12 | 28 | 30 | 3 |
| VEN125 | Venezuela | 2016 | San-Jaime | 25 | 33 | 33 | 5 |
| VEN146 | Venezuela | 2016 | Santa-Barbara | 2 | 45 | 18 | 5 |
| VEN171 | Venezuela | 2016 | Barrialito | 3 | 70 | 8 | 5 |
| K419a | Vietnam | 2015 | nd | 6 | 1 | 32 | 1 |
| K419c | Vietnam | 2015 | nd | 6 | 1 | 31 | 1 |
| K419d | Vietnam | 2015 | nd | 6 | 1 | 32 | 1 |

^a^ nd, no data. The strains are available upon request to B. Szurek at IRD.
