# Supplementary material for: A minisatellite-based MLVA for deciphering the global epidemiology of the bacterial cassava pathogen Xanthomonas phaseoli pv. manihotis
Source: PLoS One. 2023 May 11;18(5):e0285491. doi: 10.1371/journal.pone.0285491 (PMC10174486; doi:10.1371/journal.pone.0285491)
Supplement: S2 Table — (DOCX) [file pone.0285491.s006.docx]

| Pool | TR Locus | Label | Forward Sequence 5’-3’ | Label | Sequence 5’-3’ | AT | VNTR locus nomenclature from^2,4^ |
| --- | --- | --- | --- | --- | --- | --- | --- |
| 1 | Xpm1-37 |  | CGACATCAGCAGGTGGCGAC | PET- | CGACGCAGAGACAGGCGATG | 61 |  |
|  | Xpm1-08 |  | CGACGTTTCATCGCGAGCAG | VIC- | CAGCGAGCGTCTCGTCCTCA | 61 | XaG2_106^2^ |
|  | Xpm1-15 |  | GTCTTGGCCTTGGCCCACAG | NED- | ACAAGGCCGAGGGTGAGCAG | 61 |  |
| 2 | Xpm1-18 |  | GTCCATCCTTGGCGCGACCT | 6-FAM- | CAAGAGCGCCCGGTTGAAAA | 60 |  |
|  | Xpm1-21 |  | GGGAGAAGGACGCGGACTGC | NED- | GCGAGCAGGTGCAATCGGTT | 60 | XaG1_70^2^ |
|  | Xpm1-31 | PET- | TCGCAACCGCGATACGCTTT |  | GTTCACGCTGGATCAGCCGG | 60 |  |
|  | Xpm1-35 |  | GGCGTTTTTCGGTGGGAAGC | VIC- | CTGAGCAACGCACGGCTCAG | 60 |  |
| 3 | Xpm1-06 |  | GCGATAGGCCGAGGTCACCA | 6-FAM- | CCGGCGTGCATAGATGATGC | 61 |  |
|  | Xpm1-07 |  | ACTGCCACTCGTGCGAGCAC | NED- | TGATGTTCGGCAGTGGCGAT | 61 | XaG1_65^2^ |
|  | Xpm1-38 |  | TTGCGGACGCTATGGGGAAC | VIC- | TATTGGTGACCTGCACGCGG | 61 | XaG1_58^2^ |
| 4 | Xpm1-19 |  | GCGGCGAACAGCAGTAGCGT | 6-FAM- | CCAGCAATCACCCGTCCGTC | 61 | XaG1_67^4^ |
|  | Xpm1-25 |  | ATGCTCACTCCCACGGGCAT | NED- | ACCACCGGTGCGTCCATCTC | 61 | XaG1_73^4^ |
|  | Xpm1-27 |  | ATCAAGGTATCGCGGCTGGC | PET- | AATGCATCGGCATCGCCAAC | 61 | XaG1_02^4^ |
|  | Xpm1-30 |  | GCCGTGGGCGAATACGCTTC | VIC- | CTGAAGATCGGGCCACGCAG | 61 | XaG1_29^4^ |

AT: Annealing temperature.
