# Supplementary material for: A minisatellite-based MLVA for deciphering the global epidemiology of the bacterial cassava pathogen Xanthomonas phaseoli pv. manihotis
Source: PLoS One. 2023 May 11;18(5):e0285491. doi: 10.1371/journal.pone.0285491 (PMC10174486; doi:10.1371/journal.pone.0285491)
Supplement: S3 Table — (DOCX) [file pone.0285491.s007.docx]

| Locus | # Alleles | Allelic range | Nei’s gene diversity H_E_ |
| --- | --- | --- | --- |
| Xpm 2-22 | 3 | 2 | 0.104 |
| Xpm 2-18 | 8 | 7 | 0.831 |
| Xpm 2-35 | 2 | 1 | 0.159 |
| Xpm 2-20 | 4 | 3 | 0.124 |
| Xpm 2-3 | 3 | 3 | 0.551 |
| Xpm 2-23 | 4 | 3 | 0.319 |
| Xpm 2-5 | 1 | 0 | 0.000 |
| Xpm 2-33 | 2 | 1 | 0.258 |
