# Supplementary material for: A minisatellite-based MLVA for deciphering the global epidemiology of the bacterial cassava pathogen Xanthomonas phaseoli pv. manihotis
Source: PLoS One. 2023 May 11;18(5):e0285491. doi: 10.1371/journal.pone.0285491 (PMC10174486; doi:10.1371/journal.pone.0285491)
Supplement: S4 Table — (DOCX) [file pone.0285491.s008.docx]

|  | Allelic richness | Gene diversity |
| --- | --- | --- |
| Locus | A | H_E_ |
| VNTR-37 | 14 | 0.907 |
| VNTR-08 | 30 | 0.961 |
| VNTR-15 | 17 | 0.923 |
| VNTR-18 | 13 | 0.866 |
| VNTR-21 | 12 | 0.828 |
| VNTR-31* | 11 | 0.861 |
| VNTR-35 | 16 | 0.911 |
| VNTR-06* | 5 | 0.742 |
| VNTR-07* | 7 | 0.762 |
| VNTR-38* | 8 | 0.819 |
| VNTR-19 | 15 | 0.897 |
| VNTR-25 | 16 | 0.915 |
| VNTR-27 | 12 | 0.894 |
| VNTR-30 | 13 | 0.853 |

* Locus retained in the MLVA-12 scheme.
