# Supplementary material for: A minisatellite-based MLVA for deciphering the global epidemiology of the bacterial cassava pathogen Xanthomonas phaseoli pv. manihotis
Source: PLoS One. 2023 May 11;18(5):e0285491. doi: 10.1371/journal.pone.0285491 (PMC10174486; doi:10.1371/journal.pone.0285491)
Supplement: Attachment — Submitted filename: Response to Reviewers.DOC.docx [file pone.0285491.s009.docx]

March 8th, 2023

Tushar Shaw
Academic Editor
PLOS ONE

We received the reviewer´s comments on the manuscript entitled ‘A minisatellite-based MLVA for deciphering the global epidemiology of the bacterial cassava pathogen *Xanthomonas phaseoli* pv. *manihotis’*. We truly appreciate the comments, and we found them all very helpful.

We are sending a revised version of the paper where changes have been done considering their comments. Modifications in the manuscript were included with track changes mode. The response to the reviewers’ comments appear below.

Response to reviewer(s)' Comments:

Journal Requirements:

1. Please ensure that your manuscript meets PLOS ONE's style requirements, including those for file naming. The PLOS ONE style templates can be found at 
https://journals.plos.org/plosone/s/file?id=wjVg/PLOSOne_formatting_sample_main_body.pdf and 
https://journals.plos.org/plosone/s/file?id=ba62/PLOSOne_formatting_sample_title_authors_affiliations.pdf

*Response: Thanks for recommendations, the manuscript was adjusted according to PLOS ONE´s style requirements.*

2. We note that this study relies on the analysis of worldwide collection of 93 Xpm strains isolated over a period of fifty years. For reproducibility purposes, please provide the references in Supp Table 1 of all the isolates (if already published) and/or please provide further information about how others may be able to access the strains. If available on a database, please specify which one.

*Response: We included this sentence:* *“The strains are available upon request to B. Szurek at IRD”.*

3. We note that the grant information you provided in the ‘Funding Information’ and ‘Financial Disclosure’ sections do not match.

When you resubmit, please ensure that you provide the correct grant numbers for the awards you received for your study in the ‘Funding Information’ section.

*Response: Thank you, the corrected information was included in the resubmitted version.*

4. Thank you for stating the following financial disclosure:

"We thank the Faculty of Sciences from Universidad de los Andes-Bogotá, Colombia (INV-2021-128-2283), the UMR Interactions Plantes Microorganismes Environnement, and the Agropolis Foundation (project PAIX, grant no. 1 403-073), Montpellier, France, for financial support. The Ecos Nord programme supported inter-laboratory mobility (grant no. C15A01). Leidy Rache was supported by Colciencias with a doctoral fellowship (call No. 528, 2011). Olivier Pruvost acknowledges the European Union (ERDF contract GURDT I2016‐1731‐0006632) and Réunion regional council for support."

Please state what role the funders took in the study.  If the funders had no role, please state: "The funders had no role in study design, data collection and analysis, decision to publish, or preparation of the manuscript." If this statement is not correct you must amend it as needed.

Please include this amended Role of Funder statement in your cover letter; we will change the online submission form on your behalf.

*Response: the sentence “The funders had no role in study design, data collection and analysis, decision to publish, or preparation of the manuscript" was included.*

5. Please remove any funding-related text from the manuscript and let us know how you would like to update your Funding Statement. Currently, your Funding Statement reads as follows:

"We thank the Faculty of Sciences from Universidad de los Andes-Bogotá, Colombia (INV-2021-128-2283), the UMR Interactions Plantes Microorganismes Environnement, and the Agropolis Foundation (project PAIX, grant no. 1 403-073), Montpellier, France, for financial support. The Ecos Nord programme supported inter-laboratory mobility (grant no. C15A01). Leidy Rache was supported by Colciencias with a doctoral fellowship (call No. 528, 2011). Olivier Pruvost acknowledges the European Union (ERDF contract GURDT I2016‐1731‐0006632) and Réunion regional council for support."

Please include your amended statements within your cover letter; we will change the online submission form on your behalf.

*Response: As suggested, we did not include any funding statement within the manuscript. Please use the current version of the funding statement for publication.*

6. PLOS ONE now requires that authors provide the original uncropped and unadjusted images underlying all blot or gel results reported in a submission’s figures or Supporting Information files. This policy and the journal’s other requirements for blot/gel reporting and figure preparation are described in detail at https://journals.plos.org/plosone/s/figures#loc-blot-and-gel-reporting-requirements and https://journals.plos.org/plosone/s/figures#loc-preparing-figures-from-image-files. When you submit your revised manuscript, please ensure that your figures adhere fully to these guidelines and provide the original underlying images for all blot or gel data reported in your submission. See the following link for instructions on providing the original image data: https://journals.plos.org/plosone/s/figures#loc-original-images-for-blots-and-gels. 
  
In your cover letter, please note whether your blot/gel image data are in Supporting Information or posted at a public data repository, provide the repository URL if relevant, and provide specific details as to which raw blot/gel images, if any, are not available. Email us at plosone@plos.org if you have any questions.

*Response: We included the original image of an agarose gel showing the amplification results for the multiplex PCR reactions in Supporting information “Fig. S4”*

7. We note that you have included the phrase “data not shown” in your manuscript. Unfortunately, this does not meet our data sharing requirements. PLOS does not permit references to inaccessible data. We require that authors provide all relevant data within the paper, Supporting Information files, or in an acceptable, public repository. Please add a citation to support this phrase or upload the data that corresponds with these findings to a stable repository (such as Figshare or Dryad) and provide and URLs, DOIs, or accession numbers that may be used to access these data. Or, if the data are not a core part of the research being presented in your study, we ask that you remove the phrase that refers to these data.

*Response: Thanks for the suggestion, the phrase was removed because the data are not a core part of the research.*

8. We note that you have referenced (ie. Rache L, Blondin L, Flores C, Trujillo C, Szurek B, Restrepo S, et al. [40]) which has currently not yet been accepted for publication. Please remove this from your References and amend this to state in the body of your manuscript: (ie “Rache L. et al. [Unpublished]”) as detailed online in our guide for authors
http://journals.plos.org/plosone/s/submission-guidelines#loc-reference-style "

*Response: the paper is published already and can be accessed in this page: https://apsjournals.apsnet.org/doi/10.1094/PHYTO-06-18-0210-R.*

9. Please upload a new copy of Figure 3 as the detail is not clear. Please follow the link for more information:
https://blogs.plos.org/plos/2019/06/looking-good-tips-for-creating-your-plos-figures-graphics/
https://blogs.plos.org/plos/2019/06/looking-good-tips-for-creating-your-plos-figures-graphics/

*Response: A new version of Figure 3 was uploaded. We hope that this new version meets the requirements.*

10. Please review your reference list to ensure that it is complete and correct. If you have cited papers that have been retracted, please include the rationale for doing so in the manuscript text, or remove these references and replace them with relevant current references. Any changes to the reference list should be mentioned in the rebuttal letter that accompanies your revised manuscript. If you need to cite a retracted article, indicate the article’s retracted status in the References list and also include a citation and full reference for the retraction notice.

*Response: The reference list was reviewed, this is complete and correct.*Principio del formulario

Final del formulario

**Reviewer:**

1) Line 171: « 3 loci in each multiplex » but in table 1: only 2 loci are present in Pool II. So one loci is missing in table 1, Pool II. This pool is correct in Figure 2.

*Response: The reviewer is correct, locus 2-29 was included in table 1.*

2) Line 168-170: primers were tested with DNA from 9 strains (4 from Columbia, 1 from Venezuela, 1 from Ivory Coast, 1 from Burkina, 2 from Mali). Lines 170-170: these strains are not all in the S1 table. Why? if they have been tested…

*Response: The reviewer is correct, these strains are not in the S1 table because strains were used only to test the quality and design of the primers in agarose gels and they were not genotyped with labelled primers in a capillary electrophoresis system. Therefore, if we include them in table S1, they would not have comparable data. We included an extra sentence in this section: “*These strains were only used to test the quality and design of the primers in agarose gels”.

- Line 195: Xpm 2-20 is not in [40], it’s Xpm 1-20. Modify sentence

*Response: The reviewer is correct, we included an extra sentence in the manuscript:(**VNTR Xpm 1-20 nomenclature from [40]). Also, in table 2 was included “*or Rache et al [40]”

- Line 237: « 11 loci were selected » but only 10 loci are in Table 2. Modify sentence

*Response: “11 loci were selected” was replaced for “Ten loci were selected”*

- Line 253-254 : « for loci Xpm1-29 and Xpm1-2 »: is it not rather Xpm2-29 and Xpm2-2 (from Table 2) ?.

*Response: The reviewer is correct, we have now changed the numbers to “*Xpm2-29 and Xpm2-2.

- Line 262-265 : it seem's that the names of the loci are wrong, please replace 1 with 2.

*Response: The reviewer is correct, we have now changed the numbers in all instances.*

*-* Line 311 : « Four VNTRs among the least discriminant of the MLVA-14 scheme were combined » : please name them in the sentence.

*Response: the sentence “*Four VNTRs (2-31,2- 6, 2-7, 2-38) among the least discriminant of the MLVA-14 scheme were combined to the eight VNTRs (2-22, 2-18, 2-35, 2-20, 2-3, 2-23, 2-5, 2-33)…” was included

- Table 2: please explain what means the number between brackets after the motif sequence and why some letters are in bold for Xpm 2-2

*Response: The explanation was included “number of times that motif sequence is present”. Letters in bold were a mistake in format. This mistake was corrected eliminating bold.*

Reviewer:

The authors could add a figure including disease symptoms on resistant, moderately resistant, and moderately susceptible cultivars.

*Response: Thanks for the suggestion, the Figure (S3 Fig) was included.*
